# The Effects of Combined Contrast Heat Cold Pressure Therapy on Post-Exercise Muscle Recovery in MMA Fighters: A Randomized Controlled Trial

**DOI:** 10.5114/jhk/190220

**Published:** 2024-09-26

**Authors:** Robert Trybulski, Aleksandra Żebrowska, Marta Bichowska-Pawęska, Adrian Kużdżał, Ireneusz Ryszkiel, Rui Miguel Silva, Jarosław Muracki, Adam Kawczyński

**Affiliations:** 1 Medical Department Wojciech Korfanty, Upper Silesian Academy, Katowice, Poland.; 2 Provita Żory Medical Center, Żory, Poland.; 3 Department of Physiological and Medical Sciences, Institute of Sport Science, The Jerzy Kukuczka Academy of Physical Education in Katowice, Katowice, Poland.; 4 Institute of Healthy Living, The Jerzy Kukuczka Academy of Physical Education in Katowice, Katowice, Poland.; 5 Faculty of Physical Education, Gdansk University of Physical Education and Sport, Gdansk, Poland.; 6 Institute of Health Sciences, College of Medical Sciences, University of Rzeszow, Rzeszów, Poland.; 7 Department of Descriptive and Topographic Anatomy, Medical University of Silesia, Katowice, Poland.; 8 Research Center in Sports Performance, Recreation, Innovation and Technology (SPRINT), Melgaço, Portugal.; 9 Escola Superior Desporto e Lazer, Instituto Politécnico de Viana do Castelo, Viana do Castelo, Portugal.; 10 Institute of Physical Culture Sciences, Department of Physical Culture and Health, University of Szczecin, Szczecin, Poland.; 11 Department of Paralympics Sports, Wroclaw University of Health and Sport Sciences, Wrocław, Poland.

**Keywords:** Mixed Martial Arts, PPT, perfusion, myotonometer, Game Ready, fighters, muscle pain

## Abstract

The purpose of this study was to evaluate the effects of contrast heat and cold pressure therapy (CHCP) on muscle tone, elasticity, stiffness, perfusion unit, and muscle fatigue indices after plyometric training consisting of five sets of jumping on a 50-cm high box until exhaustion. A prospective, randomized, controlled single-blind study design was used. Twenty professional MMA fighters were included in the study. The experimental group (n = 10) was subjected to the CHCP protocol (eGR), while the control group (cGR) (n = 10) was subjected to sham therapy. Both protocols consisted of three CHCP sessions performed immediately after plyometric exercise, 24 and 48 h afterwards. Measurements were taken at the following time points: 1) at rest; 2) 1 min post-exercise; 3) 1 min post-CHCP therapy; 4) 24 h post-CHCP therapy; 5) 48 h post-CHCP therapy. The results of the eGR compared to the cGR showed significantly higher perfusion at time point 5 (p < 0.001), higher muscle tone at time points 1, and 3–5 (p < 0.001 for all), higher stiffness at time points 1, 3–5 (p < 0.001 for all) and a higher pain threshold at time points 1 and 5 (p < 0.001 for all). This study suggests a positive effect of CHCP therapy on muscle biomechanics, the pain threshold, and tissue perfusion, which may contribute to increasing the effectiveness of post-exercise muscle recovery in MMA athletes.

## Introduction

Excessive strain on skeletal muscles can cause mechanical damage to cell membranes, activation of inflammatory processes, increased muscle soreness, as well as increased stiffness, elasticity, and muscle tone (Enoka and Duchateau, 2008; Kelly et al., 2021). Mixed Martial Arts (MMA) fighters are highly loaded (RPE ranging from 9 to 10 in the Borg’s scale during both training and competition) in their routine training and often suffer post-exercise pain. According to Bueno et al. (2022), MMA fighters suffer pain and injuries due to training and official fights. Post-exercise muscle pain is often assessed using subjective scales or algometers. The literature shows that the Wingate test and the RSI (Reactive Strength Index) are the most frequently used tools for measuring power and anaerobic capacity (Plush et al., 2021). In this case, evaluating recovery methods that affect biomechanical and viscoelastic properties is crucial (Hansen et al., 2010; Peñailillo et al., 2015). If the muscle tone is high at rest, it may result in high intramuscular pressure, causing faster muscle fatigue and susceptibility to injury (Al-Mulla et al., 2011). MMA differ in the level and the type of training loads from other combat sports while being a comprehensive combination of many martial arts. Changes in the neuromuscular system's activity have influence on muscle strength and condition (Andrade et al., 2019; Zebrowska et al., 2019). The neuromuscular function may be impaired due to intense exercise, which may persist for several days (Moore et al., 2023), and this impairment is primarily a consequence of muscle damage and the associated inflammatory response (Cheung et al., 2003). Therefore, strategies to alleviate the adverse effects of muscle damage and inflammation by stimulating microcirculation using contrast therapy are recommended (Cholewa et al., 2016; Ravier et al., 2022; Richey et al., 2022).

Using various cold and heat therapies in recovery procedures in sports is a common practice among athletes and coaches of various disciplines, including MMA (Bueno et al., 2022; César et al., 2021; Lindsay et al., 2017; Mynarski et al., 2015; Sousa et al., 2024; Wu et al., 2023). Usually therapies using heat/cold and pressure last between 20 and 30 min (Diouf et al., 2018). In our pilot study, we estimated that 20 min were sufficient to achieve changes in muscle pain, stiffness, and tissue congestion. The temperature used in such protocols is usually 3–4 degrees Celsius for the cold stimulus and 43–45°C for the heat stimulus. Pressure, on the other hand, ranges from 5 to 75 mmHg (Alexander et al., 2021). However, very few studies have compared the effects of contrast therapy. There are no such randomized controlled trials (RCTs) in the literature, therefore, variables and characteristics of the most effective recovery protocol remain unknown. Moreover, no studies have evaluated biomechanical changes in muscles in relation to the duration of therapy (Diouf et al., 2018; Priego-Quesada et al., 2021). Most studies concern the effects of contrast therapy on the muscular system using warm-cold baths, suggesting their positive effects on post-exercise recovery, i.e., reducing muscle soreness and helping in recovery of muscle flexibility and power (Ambroży et al., 2021; Machado et al., 2016; Moore et al., 2023; Vaile et al., 2007).

The scientific literature suggests that using compression enhances the effect of heat or cold therapy (Abaïdia et al., 2017; Draper et al., 2020; Kim et al., 2021). The use of intermittent compression allows for increased tissue pressure, which accelerates reabsorption in the microcirculatory system, thereby accelerating the transport of metabolic products and oxidative stress substrates (Zebrowska et al., 2019). Compression enhances anti-inflammatory effects and can reduce muscle pain after exercise (Zuj et al., 2021). Despite the widespread use of intermittent compression (Wiśniowski et al., 2022) with emphasis on its role in the alleviation of post-exercise muscle pain (Cranston and Driller, 2022), there is no clear evidence to support the benefits of this type of treatment combined with the heat or cold therapy (Draper et al., 2020). Of interest is also a study by Wiecha et al. (2021) where it was suggested that intermittent pneumatic compression did not reduce markers of muscle damage and did not alleviate post-exercise muscle pain.

With the increasing popularity of contrast therapy, clinicians, practitioners, and athletes have sought accessible and quick-to-use portable alternatives to contrast therapy. An example of such therapy is Game Ready (GR) (www.gameready.com, USA), which can be used as a local monotherapy with heat or cold, contrast therapy or all three combined (Allan et al., 2022; Dupont et al., 2017). GR combines simultaneously the use of an alternating hot and cold stimulus that is applied to a specific body area in the form of a pressure cuff, which makes it a time-saving procedure. The pressure we can actively apply is variable, ranging from 15 to 75 (mmHg) (2–10 kPa), and the temperature is from 3 to 45°C. The duration of this treatment varies from 10 to 30 min (Alexander et al., 2021; Priego-Quesada et al., 2021). GR therapy combines three stimuli in one session: heat, cold, and compression.

The scientific literature has documented the relationship between tissue physiology and the post-stimulus response of muscles to alternating heat-cold stimuli (Alexander et al., 2021; AlSabagh et al., 2022). The most crucial physiological variable highlighted by the authors includes changes in the microvascular blood flow (AlSabagh et al., 2022; Selkow et al., 2013). The role of microcirculation has documented effects on recovery in MMA (Zebrowska et al., 2019). Other important muscle variables that are altered during hot and cold stimulation are biomechanical properties such as muscle tone, elasticity and muscle stiffness (Colantuono et al., 2023; Goswami et al., 2022; Szabo et al., 2022). These changes significantly affect athletes’ ability to undertake subsequent physical efforts and are vital to injury prevention in sport (Bahr and Holme, 2003).

By analyzing the scientific literature, we discovered gaps in assessing the impact of Game Ready contrast therapy on muscle biomechanical variables and their correlation with tissue hyperemia. The results of our study may help practitioners more effectively use the recovery protocol adopted in our research. The innovation of the study lies in the assessment conducted immediately after applying the method and over several days.

The main aim of our study was to assess the usefulness of contrast heat cold pressure (CHCP) therapy in post-exercise muscle recovery in MMA fighters. It was hypothesized that CHCP therapy would accelerate the recovery process. To investigate the return to baseline values, the following factors were analysed: muscle pain as measured by PPT, biochemical markers, i.e., LDH activity (lactate dehydrogenase), physiological variable of perfusion (PU), biomechanical muscle variables: elasticity, stiffness, and tone. The RSI was used to analyse the muscles' reactive strength which is described as the individual's capability to quickly change from an eccentric muscular contraction to a concentric one.

## Methods

### Study Procedures

**Figure 1:**
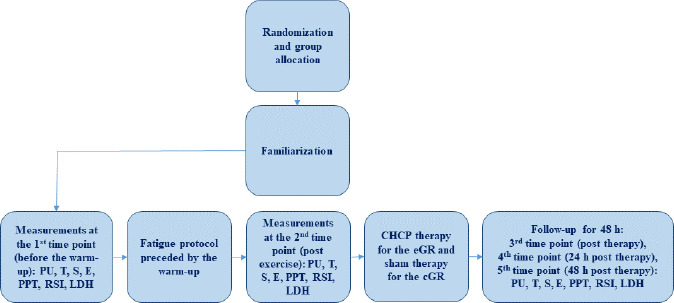
Study design.

**Figure 2:**
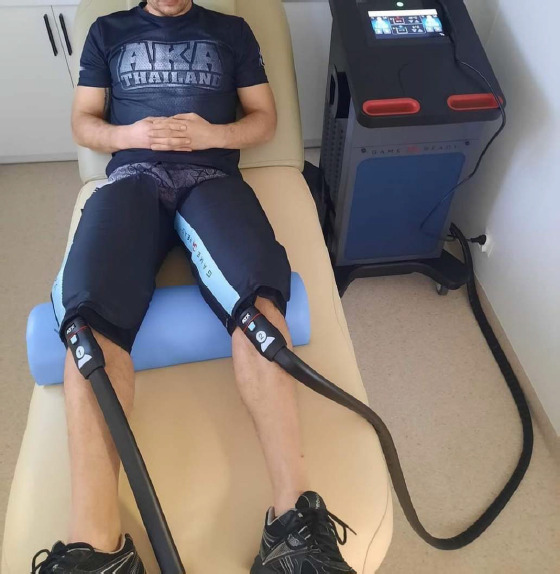
One of the participants with Game Ready therapy equipment on.

An experimental prospective, single-blind, randomized controlled trial design was applied in this study. The sample was allocated to two groups. All participants were randomly assigned (simple 1:1 randomization using randomizer.org) to an experimental (n = 10) or a control (n = 10) group before the initial assessment. The experimental group (eGR) was subjected to CHCP therapy with 20-min exposure time, while a control group (cGR) used sham GR therapy. The study design is presented in Figure 1. The sham GR procedure included the equipment sleeve that was used for 20 min (likewise the eGR), but with the system operating at minimal adjustments. Participants were blinded to the treatment and were not aware whether the group they belonged to was experimental or control. The CHCP intervention and the sham GR intervention were performed in the same way for both groups. The protocol used was in accordance with the GR manufacturer guidelines (Alexander et al., 2021). CHCP therapy was applied immediately after exercise. The participant was in a relaxed lying position with the cuffs on both legs (thighs) (Figure 2). Ultrasound-guided measurements (USG - SONOSCAPE E2, China) were taken to localize the widest cross-sectional area of the rectus femoris muscle (RF) and the medial head of the quadriceps femoris (vastus medialis, VM) in both legs. The performed measurements included: muscle tone (T [Hz]), dynamic stiffness (S [N/m]), elasticity (E [arb: relative arbitrary unit]), pressure pain threshold (PPT [N/cm]), microvascular response described in non-reference units (PU: perfusion unit), Reactive Strength Index (RSI [m·s^−1^]), and lactate dehydrogenase (LDH [U/L]) activity. The RCT was registered in the ISRCTN registry under the number ISRCTN90040217. The study was approved by the ethics committee of the National Council of Physiotherapists (approval code: 9/22; approval date: 6 April 2022) and conducted in accordance with the Declaration of Helsinki.

### Participants

Twenty young, healthy male MMA professional fighters (age: 25.5 ± 4.5 years, BMI: 24.95 ± 3.0 kg/m^2^, training experience: 10.8 ± 4.9 years) volunteered for this study. They met the following inclusion criteria: (i) age 18–40 years old; (ii) minimum three years of MMA training experience; (iii) training at least four times per week. Taking into account McKay's participant classification scheme, study participants belonged to Tier 3: highly trained/national level (McKay et al., 2022). Exclusion criteria were as follows: (i) elevated blood pressure prior to the study (blood pressure > 140/90 mm Hg); (ii) currently treated injuries, damaged skin or unspecified skin lesions at the measurement sites; (iii) a tattoo at the measurement site (as it interfered with tissue perfusion measurements); iv) taking any medications including painkillers. Athletes were also excluded in the event of extreme fatigue, fever, infection or at the explicit request of the participant at any time of the study (Allan et al., 2022). Written informed consent was obtained from participants after they were informed about the study conditions. Participants were required to abstain from training 24 h before and 48 h during the study. In addition, due to tissue perfusion measurements, participants were asked to refrain from consuming any ergogenic beverages (a list with excluded products was delivered to participants) for 4 h before the study.

### Randomization and Blinding

Group allocation was achieved through simple 1:1 randomization using a randomized sequence generated on the randomizer.org website. The randomization process was independent of treatment time and study personnel. Additionally, randomization determined the session in which each participant received either therapy (eGR) or sham therapy (cGR). One week before the study commencement, all participants completed a familiarization session including 10-min GR stimulation and the fatigue protocol. The experimental intervention involved Game Ready (GR) therapy, with each session lasting 20 min. The study was conducted by professional physicians familiar with the usage of GR. The statistician responsible for data analysis remained unaware of the person allocation throughout the study.

### Interventions

#### Contrast Heat Cold Pressure Therapy Protocol

The CHCP therapy interventions (eGR) utilized a device with cuffs placed on both thighs, which provided alternating stimulation for two minutes with cold at 3°C and pressure at 75 mmHg (10 kPa), followed by two minutes with heat at 45°C and compression at 25 mmHg (3,33 kPa) (Figure 2). For the control group (cGR) receiving sham therapy, the same procedure was followed with 2 min of warm and 2 min of cold stimulus. The total therapy duration was 20 min, with temperature of 15°C for the cold stimulus (highest GR adjustment possible) and pressure of 15 mmHg (lowest GR adjustment possible), and 36°C for the warm stimulus (neutral stimulus) with pressure of 15 mmHg. The chosen adjustments were most intensive for the eGR and least intensive for the cGR to produce the greatest possible effect, based on the assumption that the body's reaction is proportional to the applied stimulus. The picture documenting the intervention is presented in Figure 2.

#### Exercise Intervention

Although there are a limited number of studies that focused on training practices and guidelines for MMA athletes, plyometric training is a method frequently reported in the literature (Amtmann, 2004; Kostikiadis et al., 2018; La Bounty et al., 2011; Tack, 2013).

**Figure 3:**
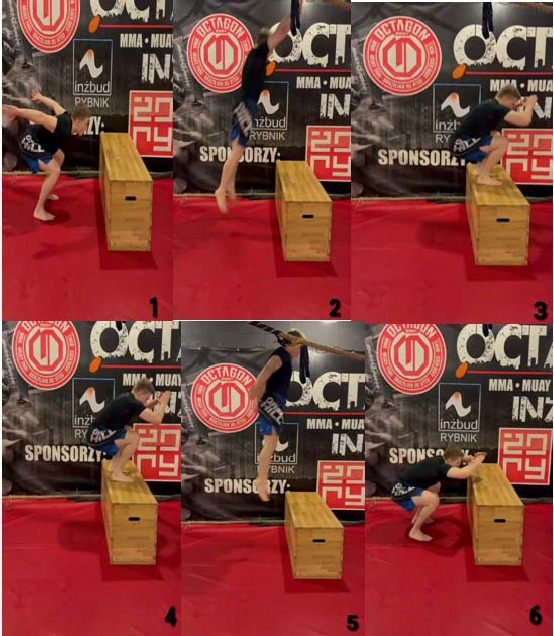
Box jumps execution: 1. Initial phase: position in front of a plyometric box (lowering their bodies by flexing their hips, knees, and extension of their ankles and arms); 2. Jump phase (concentric), a dynamic jump up and forward (extension of their hips, knees, and flexing ankles and arms); 3. Loading response phase on the box (eccentric); 4. Initial phase of the drop jump; 5. Dynamic drop jump; 6. Loading response phase on the ground.

As described by Loturco et al. (2023) box jumps are widely used in several sports and among different age categories. Many researchers recommend the use of box jumps mainly because they allow the reduction in ground reaction forces when landing, thus limiting the increased risk of injury commonly associated with excessive eccentric loads (Booth and Orr, 2016; Fort-Vanmeerhaeghe et al., 2016; Prapavessis et al., 2003). Due to the multifactorial nature of MMA, it is challenging to develop a comprehensive assessment of combat sports performance. However, it is possible to assess selected physiological and physical characteristics contributing to competitive success. Plush et al. (2021) described tests for MMA fighters, including elements of plyometric training in the form of the countermovement jump (CMJ). Therefore, box jumps were used in this study. The protocol inducing fatigue and muscle damage consisted of 4–6 sets of plyometric jumps on a 50-cm high box (Figure 3) until the participant was unable to continue the effort. There were 20-s rest intervals between successive sets. Before the fatigue task, participants performed a warm-up comprising 5-min cycling on a bicycle ergometer with moderate intensity and 3-min lower limbs stretching (including knee flexors and extensors, hip abductors and adductors and triceps surae). Throughout the fatigue protocol, participants were supervised by a paramedic.

#### Reactive Strength Index

The RSI was used to assess lower limb muscles’ reactive strength. The reliability of measures of reactive force and related variables of kinematic efficiency obtained during drop jumps has been previously confirmed (Healy et al., 2018). The measurement was made using the Force Decks ground reaction force plate (Vald-Performance, Australia, 2012). The RSI describes an individual's ability to change from eccentric to concentric muscle contraction quickly and is intended to assess an athlete's reactive strength. Participants had to jump down from a 50 cm high box and make a maximum vertical jump after landing on the force plate. In time points 1, 4 and 5 prior to the test, participants performed passive lower limbs stretches for one minute in total, 2 sets of 3 free jumps and 1 set of 10 sit-ups. For time points 2 and 3 there was no additional warm-up because participants were already after the exercise protocol at these time points. All athletes were instructed in the jumping technique by a research assistant and made three attempts before taking measurements (Markwick et al., 2015). The best result was used in further analysis. The following formula was used for the RSI calculation: RSI = Jump Height/Contact Time (m·s^−1^) (Flanagan et al., 2008).

#### Tissue Perfusion Unit (PU)

Perfusion was measured first at rest and after stimulation. Tissue perfusion was analysed with a Laser Doppler Flowmeter (LDF) using the Perimed device (Sweden, 2004). LDF is the gold standard in assessing microcirculatory responses, demonstrating high sensitivity and repeatability of measurements (Kvandal et al., 2006). The wave reflected from the erythrocytes was recorded at a skin tissue volume of 1 mm^3^ and a depth of 2.5 mm. The LDF method, because of its reproducibility, high sensitivity, and non-invasiveness, allows precise assessment of the microcirculatory response to physical stimuli or post-stimulus response (Roberts et al., 2017). The well-established standardisation of measurements was used (Liana et al., 2009). The measurement time was 2 min. Some authors suggest that hyperaemic responses observed in the skin can be interpreted as changes in muscle tissue (Choi et al., 2014; Eriksson et al., 1986; Poole, 2019). During tissue perfusion testing, PU(r) (resting flow) and PU(p) (post-stimulation hyperaemia) are most commonly determined. These ratios have no values or reference units and are referred to as perfusion units (Szyguła et al., 2020).

#### Myotonometry

Myotonometry was performed as a second measurement at rest and after stimulation. Measurements were taken with a myotonometer (MyotonPRO AS, Myoton Ltd, Estonia 2021). Myoton is a digital device consisting of a device body and a depth probe (Ø 3 mm). Through the probe, pre-pressure (0.18 N) is applied to the surface, which compresses the underlying material. A mechanical impulse (0.4 N, 15 ms) is then released through the device, which deforms the medium for a short time. Myotonometry is a reliable measurement method and can detect differences in physical properties compared to stretched muscle fibres (Bartsch et al., 2023; Melo et al., 2022). The measurement method consists of registering the damped natural vibrations of soft biological tissue in the form of an acceleration signal and then simultaneously calculating the variables of the state of stress and biomechanical properties: muscle tension (T [Hz]), stiffness (S [N/m]), and elasticity (E [arb: relative arbitrary unit]) (Bartsch et al., 2023). These measurements were performed on the most salient points of the muscle belly of the vastus medialis (VM) and the rectus femoris (RF) as proposed by Chen et al. (2019) and the methodology was in accordance with the manufacturer’s directions. The midpoint of the rectus femoris was determined in the supine position with a foam roller under the knee with a tape measure applied on the line formed between the patella's upper edge and the pelvis's iliac spine. Since the area of the medial head of the quadriceps femoris muscle was small, a measuring tape was not used, but the ultrasound control of the widest section of the muscle was applied (Muckelt et al., 2022).

#### Pressure Pain Threshold

The pressure pain threshold (PPT [N/cm]) measurement was performed as the third of all measurements at rest and after stimulation. The PPT was measured using the FPIX algometer (Wagner Instruments, Greenwich, CT, USA, 2013). The determination of the pressure pain threshold is an attempt to objectively control the pain threshold (Park et al., 2011). These measurements were performed on the vastus medialis (VM) and rectus femoris (RF) in accordance with the manufacturer’s directions (Wagner Instruments). Participants were subjected to a probe (r = 4 mm) compression test three times, inducing compressive forces in a defined area, which was marked and did not change during the study. The value of force [N/cm] was digitally displayed on the screen and calculated as the average of the three measurements. The pressure was applied until the test stimulus was signalled as unpleasant.

#### Lactate Dehydrogenase Activity

Serum enzyme activity blood samples (8 ml total) were collected from the cubital vein in a sitting position by the venepuncture method into Innmedis (Poland) clotting tubes by qualified personnel. The amount was 8 ml as there were also other variables investigated, yet they were not included in this article. The tubes were immediately sent to the laboratory where the blood was centrifuged (3000 g for 10 min at 4°C) using a centrifuge (MPW −54). The serum was transferred to the tubes and stored at −80°C until analysis. A certified laboratory diagnostician analysed all samples to minimise the impact of inter-assay variability. The enzyme activity was analysed in triplicate in the spectrometer. Dehydrogenase activity was determined in the serum by colorimetric spectrophotometry at 340 nm and 37°C using ready-made diagnostic reagents (Alpha Diagnostic, Poland) in an A15 automatic biochemical analyser (Randox, Polska).

All measurements were taken under standardized conditions in a resting semi-sitting position on a medical chair with a flexion angle of 20 degrees and leaning against the chair, between 9 a.m. and 12 p.m. (Markwick et al., 2015; Roberts et al., 2017). The measurements were taken at the following time points: 1) at rest: before the warm-up preceding exercise; 2) post-exercise: 1 min after exercise; 3) post-CHCP therapy: 1 min after CHCP therapy; 4) 24 h post-CHCP therapy; 5) 48 h post-CHCP therapy. The evaluations were taken in the following order: 1) PU, 2) T, 3) S, 4) E, 5) PPT, 6) RSI, and 7) LDH. Participants used no other recovery methods, and their dietary habits remained unchanged. All participants were informed that they should not participate in any additional physical activity except this implemented in the study and were instructed to have a minimum of 8 h of sleep.

### Statistical Analyses

G*Power software (version 3.1.9.2; Kiel University, Kiel, Germany) (Faul et al., 2007) was used to estimate the required sample size setting a minimum expected effect size of at least 0.4 (Bieuzen et al., 2013), an α level of 0.05, power (1–β) of 0.8, correlation among repeated measures of 0.5 and statistical test: ANOVA, repeated measures within-between interaction. The procedure required a minimum number of 8 participants per group. Considering participants drop-out, initially there were 22 participants recruited, yet two were excluded from the study finally giving 10 participants in each of the two groups (n eGR = 10, n cGR = 10, n = 20 total).

Results are presented as means and standard deviations. The Shapiro-Wilk, Levene and Mauchly’s tests were used in order to verify the normality, homogeneity and sphericity of the sample data variances, respectively. Differences between the therapy and control conditions were examined using repeated measures three-way ANOVA (2 conditions [therapy vs control] × 2 muscle [vastus medialis vs. rectus femoris] × 5-time points [1^st^ vs. 2^nd^ vs. 3^rd^ vs. 4^th^ vs. 5^th^]). Effect sizes for main effects and interactions were determined by partial eta squared (η_p_^2^). Partial eta squared values were classified as small (0.01 to 0.059), moderate (0.06 to 0.137), and large (> 0.137). Post hoc comparisons using the Tukey’s test were conducted to locate the differences between mean values when a main effect or an interaction was found. For pairwise comparisons, ESs were determined by Cohen’s *d* which was characterized as large (*d* > 0.8), moderate (*d* between 0.8 and 0.5), small (*d* between 0.49 and 0.20), and trivial (*d* < 0.2). Percent changes with 95% confidence intervals (95CI) were also calculated. Statistical significance was set at *p* < 0.05. All data were analyzed using Statistica (version 9.1; Statsoft, Inc., Tulsa, OK, USA).

## Results

The three-way repeated measures ANOVA did not show a significant multi-interaction effect (condition vs. muscle vs. time point) for PU (*p* = 0.80; η_p_^2^ = 0.04; Table 1). Furthermore, three-way repeated measures ANOVA showed a significant interaction effect (condition vs. time point) for PU (*p* = 0.005; η_p_^2^ = 0.32) as well as a significant main effect of condition (*p* < 0.001; η_p_^2^ = 0.75). The post-hoc Tukey comparison for the main effect of the condition showed a significantly lower PU for therapy compared to the control condition (9.87 vs. 11.32 [PU]; *p* < 0.001). The post-hoc Tukey comparison for the interaction effect (condition vs. time point) showed a significantly higher PU for the therapy condition compared to the control in the 5^th^ time point (*p* < 0.001 for all).

**Table 1:** Comparisons between the experimental conditions for perfusion.

Condition	1^st^ time point [PU] (95%CI)	2^nd^ time point [PU] (95%CI)	3^rd^ time point [PU] (95%CI)	4^th^ time point [PU] (95%CI)	5^th^ time point [PU] (95%CI)
**Therapy RF**	10.7 ± 1.8 (9.4 to 12.0)	12.1 ± 1.2 (11.3 to 13.0)	11.5 ± 1.2 (10.7 to 12.3)	11.9 ± 3.6 (9.3 to 14.5)	11.2 ± 0.8* (10.7 to 11.8)
**Therapy VM**	9.9 ± 2.2 (8.4 to 11.5)	12.2 ± 1.0 (11.4 to 12.9)	12.1 ± 2.0 (10.7 to 13.5)	11.1 ± 1.6 (10.0 to 12.3)	10.5 ± 1.4# (9.5 to 11.5)
**Control RF**	9.2 ± 1.1 (8.5 to 10.0)	13.1 ± 1.8 (11.8 to 14.3)	9.9 ± 1.7 (8.7 to 11.1)	9.3 ± 2.4 (7.5 to 11.0)	8.4 ± 0.8* (7.8 to 8.9)
**Control VM**	8.5 ± 1.4 (7.5 to 9.5)	13.5 ± 2.3 (11.8 to 15.2)	9.6 ± 2.7 (7.7 to 11.6)	9.4 ± 1.9 (8.0 to 10.7)	8.0 ± 0.8# (7.5 to 8.6)
**Effect Size Cohen’s *d* comparison**					
**Therapy vs. Control RF**	1.01	0.65	1.09	0.85	3.5
**Therapy vs. Control VM**	0.76	0.73	1.05	0.97	2.19

All data are presented as mean ± SD; CI: confidence interval; VM: vastus medialis; RF: rectus femoris; results with significant differences are marked: * significant differences compared to the control RF condition; # significant differences compared to the control VM condition, p < 0.001

The three-way repeated measures ANOVA showed a significant multi-interaction effect (condition vs. muscle vs. time point) for muscle tone (*p* < 0.001; η_p_^2^ = 0.49) and a significant interaction effect (condition vs. time point) for muscle tone (*p* < 0.001; η_p_^2^ = 0.58) as well as a significant main effect of condition (*p* < 0.001; η_p_^2^ = 0.81). The post-hoc Tukey significant differences for the multi-interaction effect are presented in Table 2.

**Table 2:** Comparisons between the experimental conditions for muscle tone.

Condition	1^st^ time point [Hz] (95%CI)	2^nd^ time point [Hz] (95%CI)	3^rd^ time point [Hz] (95%CI)	4^th^ time point [Hz] (95%CI)	5^th^ time point [Hz] (95%CI)
**Therapy RF**	14.2 ± 0.9 (13.5 to 14.8)	19.5 ± 0.9 (18.8 to 20.2)	15.9 ± 0.8 (15.3 to 16.5)	15.2 ± 0.9 (14.6 to 15.9)	15.1 ± 1.1 (14.4 to 15.9)
**Therapy VM**	14.5 ± 0.8* (13.9 to 15.0)	19.3 ± 0.9 (18.6 to 19.9)	15.4 ± 0.9* (14.7 to 16.1)	15.4 ± 0.6* (14.9 to 15.8)	15.3 ± 0.9* (14.7 to 16.0)
**Control RF**	15.3 ± 1.0 (14.5 to 16.0)	18.6 ± 1.3 (17.7 to 19.6)	15.7 ± 0.7 (15.2 to 16.2)	15.7 ± 0.9 (15.1 to 16.4)	15.1 ± 0.8 (14.5 to 15.6)
**Control VM**	15.7 ± 1.0* (15.0 to 16.4)	18.4 ± 0.6 (18.0 to 18.8)	17.7 ± 1.0* (17.0 to 18.3)	17.3 ± 1.6* (16.1 to 18.4)	18.8 ± 0.8* (18.2 to 19.4)
**Effect Size Cohen’s *d* comparison**					
**Therapy vs. Control RF**	1.16	0.80	0.27	0.56	0.00
**Therapy vs. Control VM**	1.33	1.18	2.42	1.57	4.11

All data are presented as mean ± SD; CI: confidence interval; results with significant differences are marked: * significant differences compared to the control VM condition; p < 0.001; VM: vastus medialis; RF: rectus femoris

The post-hoc Tukey comparison for the main effect of the condition showed significantly higher muscle tone for therapy compared to the control condition (16.82 vs. 15.97 [Hz]; *p* < 0.001). The post-hoc Tukey comparison for the interaction effect (condition vs. time point) showed significantly higher muscle tone for the therapy condition compared to control in the 1^st^, 3^rd^, 4^th^, and 5^th^ time points (*p* < 0.001 for all).

The three-way repeated measures ANOVA did not show a significant multi-interaction effect (condition vs. muscle vs. time point) for stiffness (*p* = 0.55; η_p_^2^ = 0.07, Table 3). However, a significant interaction effect (condition vs time point) for stiffness (*p* < 0.01; η_p_^2^ = 0.28) as well as a significant main effect of condition (*p* < 0.001; η_p_^2^ = 0.74) were observed. The post-hoc Tukey comparison for the main effect of the condition showed significantly higher stiffness for the therapy compared to the control condition (330.76 vs. 292.74 [N/m]; *p* < 0.001). The post-hoc Tukey comparison for the interaction effect (condition vs. time point) showed significantly higher stiffness for the therapy compared to the control condition in the 1^st^, 3^rd^, and 5^th^ time points (*p* < 0.001 for all).

**Table 3:** Comparisons between the experimental conditions for muscle stiffness.

Condition	1^st^ time point [N/m] (95%CI)	2^nd^ time point [N/m] (95%CI)	3^rd^ time point [N/m] (95%CI)	4^th^ time point [N/m] (95%CI)	5^th^ time point [N/m] (95%CI)
**Therapy RF**	257.1 ± 31.3* (234.7 to 279.5)	358.1 ± 43.0 (327.2 to 389.0)	267.9 ± 19.0* (253.7 to 282.1)	280.7 ± 50.0 (244.8 to 316.6)	279.3 ± 30.5* (257.5 to 301.1)
**Therapy VM**	269.3 ± 35.3# (244.1 to 294.5)	341.5 ± 38.4 (314.2 to 368.8)	272.2 ± 14.5# (261.6 to 282.8)	306.0 ± 34.3 (281.1 to 330.9)	295.3 ± 22.5# (279.2 to 311.4)
**Control RF**	259.5 ± 34.2* (234.7 to 284.3)	366.1 ± 27.1 (346.4 to 385.8)	330.1 ± 44.6* (298.5 to 361.7)	323.1 ± 39.7 (294.6 to 351.6)	345.9 ± 37.6* (319.0 to 372.8)
**Control VM**	279.1 ± 37.3# (252.4 to 305.8)	370.9 ± 22.7 (354.6 to 387.2)	345.3 ± 41.7# (315.4 to 375.2)	336.5 ± 43.1 (305.7 to 367.3)	351.1 ± 36.1# (325.3 to 376.9)
**Effect Size Cohen’s *d* comparison**					
**Therapy vs. Control RF**	0.07	0.22	1.81	0.94	1.95
**Therapy vs. Control VM**	0.27	0.93	2.34	0.78	1.86

All data are presented as mean ± SD; CI: confidence interval; results with significant differences are marked: *significant differences compared to the control RF condition; # significant differences compared to the control VM condition p < 0.001; VM: vastus medialis; RF: rectus femoris

The three-way repeated measures ANOVA showed a significant multi-interaction effect (condition vs. muscle vs. time point) for elasticity (*p* < 0.001; η_p_^2^ = 0.30) and a significant main effect of condition (*p* < 0.01; η_p_^2^ = 0.52). The post-hoc Tukey comparison for the main effect of the condition showed significantly higher elasticity for the therapy compared to the control condition (1.51 vs. 1.32 [N]; *p* < 0.001). The post-hoc Tukey significant differences for the multi-interaction effect are presented in Table 4.

**Table 4:** Comparisons between the experimental conditions for elasticity.

Condition	1^st^ time point [arb] (95%CI)	2^nd^ time point [arb] (95%CI)	3^rd^ time point [arb] (95%CI)	4^th^ time point [arb] (95%CI)	5^th^ time point [arb] (95%CI)
**Therapy RF**	1.05 ± 0.07 (1.00 to 1.10)	1.69 ± 0.17 (1.57 to 1.81)	1.27 ± 0.05* (1.23 to 1.30)	1.28 ± 0.11* (1.20 to 1.36)	1.27 ± 0.10 (1.19 to 1.34)
**Therapy VM**	1.20 ± 0.09 (1.14 to 1.26)	1.55 ± 0.16 (1.44 to 1.66)	1.32 ± 0.06 (1.28 to 1.36)	1.37 ± 0.23# (1.21 to 1.54)	1.23 ± 0.11# (1.15 to 1.31)
**Control RF**	1.24 ± 0.30 (1.02 to 1.45)	1.68 ± 0.25 (1.50 to 1.85)	1.59 ± 0.26* (1.40 to 1.77)	1.67 ± 0.32* (1.45 to 1.90)	1.41 ± 0.09 (1.35 to 1.48)
**Control VM**	1.20 ± 0.33 (0.97 to 1.43)	1.70 ± 0.20 (1.56 to 1.84)	1.47 ± 0.17 (1.34 to 1.59)	1.62 ± 0.36# (1.35 to 1.88)	1.51 ± 0.24# (1.34 to 1.68)
**Effect Size Cohen’s *d* comparison**					
**Therapy vs. Control RF**	0.87	0.05	1.71	1.63	1.47
**Therapy vs. Control VM**	0	0.83	1.18	0.83	1.50

All data are presented as mean ± SD; CI: confidence interval; results with significant differences are marked: * significant differences compared to the control RF condition; # significant differences compared to the control VM condition p < 0.001; VM: vastus medialis; RF: rectus femoris

The three-way repeated measures ANOVA did not show a significant multi-interaction effect (condition vs. muscle vs. time point) for pain threshold (*p* = 0.24; η_p_^2^ = 0.13. Table 5), but a significant interaction effect (condition vs. time point) for the pain threshold (*p* < 0.001; η_p_^2^ = 0.32). The post-hoc Tukey comparison for the interaction effect (condition vs. time point) showed a significantly higher pain threshold for the therapy compared to the control condition in the 1^st^ and 5^th^ time points (*p* < 0.001 for all) (Table 5).

**Table 5:** Comparisons between the experimental conditions for the pain threshold.

Condition	1^st^ time point [N/cm] (95%CI)	2^nd^ time point [N/cm] (95%CI)	3^rd^ time point [N/cm] (95%CI)	4^th^ time point [N/cm] (95%CI)	5^th^ time point [N/cm] (95%CI)
**Therapy RF**	193.9 ± 33.1* (170.2 to 217.5)	126.7 ± 17.1 (114.5 to 138.9)	145.5 ± 29.9 (124.2 to 166.9)	152.5 ± 27.4 (132.9 to 172.1)	153.1 ± 25.0* (135.2 to 171.0)
**Therapy VM**	197.0 ± 36.1# (171.2 to 222.8)	126.8 ± 16.2 (115.2 to 138.4)	139.5 ± 38.9 (111.7 to 167.3)	150.7 ± 28.3 (130.4 to 170.9)	167.6 ± 26.0# (149.0 to 186.1)
**Control RF**	185.7 ± 38.0* (158.5 to 212.9)	138.3 ± 31.7 (115.6 to 161.0)	133.5 ± 37.9 (106.4 to 160.7)	125.2 ± 32.0 (102.3 to 148.1)	112.5 ± 18.0* (99.6 to 125.3)
**Control VM**	186.4 ± 42.7# (155.9 to 217.0)	136.7 ± 23.0 (120.3 to 153.2)	134.8 ± 36.7 (108.6 to 161.1)	127.0 ± 38.5 (99.4 to 154.5)	115.2 ± 13.6# (105.4 to 125.0)
**Effect Size Cohen’s *d* comparison**					
**Therapy vs. Control RF**	0.23	0.34	0.35	0.92	1.86
**Therapy vs. Control VM**	0.27	0.50	0.12	0.70	2.53

All data are presented as mean ± SD; CI = confidence interval; VM: vastus medialis; RF: rectus femoris, results with significant differences are marked: * significant differences compared to the control RF condition; # significant differences compared to the control VM condition (p < 0.001)

The two-way repeated measures ANOVA showed a significant interaction effect (condition vs. time point) for the RSI (*p* < 0.001; η_p_^2^ = 0.44). The post-hoc Tukey did not show differences between conditions for individual time points (Table 6).

**Table 6:** Comparisons between the experimental conditions for the RSI.

Condition	1^st^ time point [m·s^−1^] (95%CI)	2^nd^ time point [m·s^−1^] (95%CI)	3^rd^ time point [m·s^−1^] (95%CI)	4^th^ time point [m·s^−1^] (95%CI)	5^th^ time point [m·s^−1^] (95%CI)
**Therapy**	1.94 ± 0.35 (1.69 to 2.19)	1.08 ± 0.32 (0.85 to 1.31)	1.60 ± 0.26 (1.41 to 1.79)	1.74 ± 0.26 (1.55 to 1.92)	1.71 ± 0.37 (1.45 to 1.98)
**Control**	2.08 ± 0.37 (1.82 to 2.34)	1.32 ± 0.30 (1.10 to 1.53)	1.45 ± 0.28 (1.25 to 1.65)	1.55 ± 0.34 (1.30 to 1.79)	1.46 ± 0.29 (1.25 to 1.67)
**Effect Size Cohen’s *d* comparison**					
**Therapy vs. Control**	0.39	0.77	0.56	0.63	0.75

All data are presented as mean ± SD; CI = confidence interval

The two-way repeated measures ANOVA showed a significant interaction effect (condition vs. time point) for LDH (*p* = 0.009; η_p_^2^ = 0.30), although the post-hoc Tukey showed differences between conditions only in the 3^rd^ time point (Table 7).

**Table 7:** Comparisons between the experimental conditions for LDH.

Condition	1^st^ time point [U/L] (95%CI)	2^nd^ time point [U/L] (95%CI)	3^rd^ time point [U/L] (95%CI)	4^th^ time point [U/L] (95%CI)	5^th^ time point [U/L] (95%CI)
**Therapy**	182.0 ± 20.2 (167.5 to 196.5)	250.1 ± 33.1 (226.4 to 273.8)	196.7 ± 22.1* (180.9 to 212.5)	192.4 ± 13.0 (183.1 to 201.7)	176.4 ± 21.6 (160.9 to 191.9)
**Control**	184.9 ± 17.8 (172.2 to 197.6)	244.5 ± 34.5 (219.8 to 269.2)	226.1 ± 31.2* (203.8 to 248.4)	208.5 ± 31.8 (185.7 to 231.3)	191.0 ± 31.1 (168.8 to 213.2)
**Effect Size Cohen’s *d* comparison**					
**Therapy vs. Control**	0.15	0.17	1.09	0.66	0.55

All data are presented as mean ± SD; CI = confidence interval; results with significant differences are marked: * significant differences compared to control condition; p < 0.05

## Discussion

The primary aim of this study was to evaluate the recovery effect of CHCP on various biomechanical properties, including muscle tone, elasticity, stiffness, and PU, as well as markers of muscle fatigue. The main findings confirm CHCP's positive impact on increased tissue perfusion, particularly concerning flow-mediated dilation (FMD). These changes in FMD play a vital role in determining the adaptive capacity of the vascular endothelium, which, in turn, significantly influences post-exercise recovery processes (Brunt et al., 2016; Chiesa et al., 2017).

Various interventions, such as heat or cold stress, can elicit diverse haemodynamic responses and improve these physiological capacities (AlSabagh et al., 2022; Kim et al., 2020c; Ogoh et al., 2013). Kim et al. (2020a) confirmed an increased blood flow measured with the use of LDF by postulating that hyperaemic responses facilitated the recovery process by promoting nutrient delivery. It should be noted that some authors have suggested that skin perfusion as measured by LDF may have a counterpart in the responses of muscle hyperaemia (Eriksson et al., 1986; Kvandal et al., 2006). Akasaki et al. (2006) demonstrated through an animal experiment that repeated thermal therapy led to increased eNOS protein expression, an enhanced blood flow and higher capillary density in the ischaemic hind limb of mice. The endothelium plays a pivotal role in the mechanism of local microvascular autoregulation, serving as a source for various mediators. Among these, NO and prostacyclin act as potent vasodilators, while EDCF2 and endothelin (EDCF1) function as robust vasoconstrictors (Minson et al., 2001).

CHCP therapy showed a beneficial effect in regulating the biomechanical properties of muscles after exercise (Kim et al., 2020b). Muscle tone, elasticity, and stiffness are mainly maintained by the complex interplay of spinal and supraspinal mechanisms, the disruption of which can cause many changes in the biomechanical properties of muscles (Graven-Nielsen et al., 2002; Messina, 1990). It is widely acknowledged that a thermal stimulus leading to increased muscle hyperaemia can effectively reduce muscle tone while simultaneously improving muscle stiffness and elasticity (Kim et al., 2020). Although the exact mechanisms behind these effects are not fully understood, it is generally accepted that non-myogenic regulation of muscle tone, linked to enhanced perfusion, also contributes to the observed improvements. Specifically, elevated Ca^2+^ concentrations in the cytosol, resulting from impaired perfusion and subsequent tissue hypoxia, can trigger muscle contraction by activating the phosphorylation of myosin light chains and the subsequent actomyosin cross-bridging, resulting in increased tone. Conversely, the activation of the capillary system helps counteract tissue hypoxia and can lead to a reduction in muscle tone (Huxel et al., 2008; Kim et al., 2020c).

Previous research suggested that a musculotendinous system with higher elasticity (lower stiffness) has a greater capacity to elongate, enabling it to absorb external forces and create a mitigating effect on energy production during movement (Roberts and Konow, 2013). In the present study, we focused on stiffness, muscle tone, and elasticity as crucial variables influencing muscle strength, power generation, and the risk of injury in sports. It is essential to highlight that for proper energy dissipation, muscle bundles must actively elongate, maintaining the right balance of elasticity and stiffness (Nuñez et al., 2022; Roberts and Konow, 2013). Studies conducted both *in situ* and *in vivo* indicate that the tendon fibres surrounding muscles can delay this elongation during energy-dissipating events by temporarily absorbing impact energy and then releasing it to work on the muscle bundles. This intricate mechanism relies not only on appropriate neural control, but also on adequate blood distribution through microcirculation (Bieuzen et al., 2013; Cochrane, 2004). CHCP therapy appears to be one of the stimuli affecting these biomechanical properties of the muscles.

Our PPT results show CHCP's effects on reducing muscle pain, and they support the general hypothesis of the analgesic effects of contrast therapy (Colantuono et al., 2023). There is insufficient evidence in the scientific literature for the use of CHCP therapy in the treatment of post-exercise muscle pain. Researchers have mainly focused on the evaluation of use of CHCP to decrease pain in sports injuries (Diouf et al., 2018) and the assessment of hydrotherapy and cold compresses (Vaile et al., 2007; Versey et al., 2012; Wang et al., 2022). It has been shown that contrast hydrotherapy eliminates the negative effects of exercise-induced muscle damage (EIMD), inflammation and delayed onset muscle soreness (DOMS) (Bieuzen et al., 2013), while increasing the rate of strength and power recovery (Colantuono et al., 2023), and joint mobility after exercise (Cochrane, 2004). Colantuono et al. (2023) note that previous studies of contrast therapy combined with compression have not analysed the muscle recovery capacity after intense exercise or the assessment of recovery of intramuscular glycogen stores. The authors' results are thus intriguing and suggest a positive effect of CHCP on recovery after intense eccentric exercise and recovery of intramuscular glycogen stores associated with EAMD.

There is insufficient evidence regarding the effect of CHCP on enhancing the recovery process of muscle strength and power. In our study, the effect of CHCP on recovery of strength measured by the RSI was noticeable at the 3^rd^, 4^th^ and 5^th^ time points (effect size 0.56, 0.63, 0.75, respectively) which are key time periods for the post-exercise recovery process, however, differences between the eGR and the cGR were not significant when evaluatd by the Tukey’s test. Most studies have focused on analysing these variables after applying a thermal stimulus, indicating heat shock protein (HSP) activity (Kim et al., 2020a; Liu, 2001; Xie et al., 2015). HSPs are considered molecular chaperone proteins that play a universal role in maintaining cellular homeostasis. HSPs have been confirmed to be expressed in skeletal muscle, the induction of which varies according to histological and even functional muscle characteristics (Xie et al., 2015). Heat increases gene expression in muscle cell growth and differentiation (McGorm et al., 2018).

Our results confirmed faster decreases in LDH in the experimental group. It has been also claimed that the warm-cold contrast increases lactate clearance, reduces post-exercise oedema and improves the blood flow to fatigued muscles (French et al., 2008). Stadnyk et al. (2017) suggested that significant fluctuations in skin temperature caused by hot and cold contrast packs caused vasoconstriction and vasodilation, thereby initiating an increased hyperaemic response, which might be one of the mechanisms of accelerated clearance of markers of muscle fatigue (Zebrowska et al., 2019). It has been previously found that contrast bathing (CB) and compression garments (CG) did not promote faster recovery after intense training more effectively than passive conditions. However, contrast bathing temporarily relieved post-exercise soreness (French et al., 2008).

Despite the widespread use of contrast therapy in the field, evidence remains insufficient to optimise post-exercise recovery protocols. Despite searching through various scientific databases, such as the Cochrane Library, EBSCO, Google Scholar, and PubMed, using keywords such as "contrast therapy", "game ready therapy" and "contrast therapy time", no relevant or randomized controlled trial (RCT) results assessing the effects of CHCP therapy were found. As the first RCT of CHCP therapy, our study offers crucial insights, indicating the efficacy of CHCP therapy in reducing post-exercise muscle fatigue among MMA fighters.

## Limitations and Directions for Future Research

This study presents some limitations. The main limitation lies in a small sample size, yet obtaining a larger, diverse group of professional MMA fighters is difficult due to injuries and competition. Additionally, the immediate effect observed in the results may be influenced by a thixotropic effect, although its magnitude remains unclear. The LDF method used is highly sensitive and requires strict testing procedures, which could potentially distort the observed changes. It is essential to acknowledge that some individuals may strongly prefer CHCP therapy, potentially influencing the measured variables. Given the study's small sample size, future research should include a more extensive and more diverse sample with extended observation periods. Future research should also consider including more sessions with a crossover design. Comparing CHCP therapy’s effects on muscle stiffness, resting tone, and elasticity with other post-exercise recovery methods could also be a valuable focus for future projects. Moreover, exploring CHCP therapy’s effects in individuals with varying levels of physical preparation and across different sports would be beneficial. Another limitation of the research is that there is no current consensus on the best repeat effort protocol for MMA athletes with many protocols being utilized. Furthermore, the Ultimate Fighting Championship (UFC) Performance Institute (PI) does not include any repeat effort ability testing (Plush et al., 2021). Nevertheless, it is still essential to include a measure of repeated effort ability in any testing battery of MMA performance that is at least well justified theoretically until future research can investigate further (Plush et al., 2021).

## Practical Implications

Our research indicates the effectiveness of contrast therapy in post-exercise recovery, providing coaches and physiotherapists with additional tools to reduce the effects of fatigue in athletes. A single twenty-minute session of CHCP therapy seems sufficient to achieve beneficial muscle changes which optimize recovery.

## Conclusions

In conclusion, the present study highlights the significant impact of CHCP as a stimulus, influencing essential aspects of muscle biomechanics, the pain threshold, muscle strength, and tissue perfusion. With the reliable and non-invasive nature of MyotonPRO for assessing biomechanical muscle properties in MMA athletes, we unveil potential for optimising contrast therapy in sports. Despite challenges related to group size and diversity, our findings open avenues for further research and advancements, propelling contrast therapy toward unlocking greater athletic potential and enhancing recovery strategies in the world of sports performance.
